# Molecular and cellular responses of the pathogenic fungus *Lomentospora prolificans* to the antifungal drug voriconazole

**DOI:** 10.1371/journal.pone.0174885

**Published:** 2017-03-31

**Authors:** Aize Pellon, Andoni Ramirez-Garcia, Idoia Buldain, Aitziber Antoran, Aitor Rementeria, Fernando L. Hernando

**Affiliations:** Fungal and Bacterial Biomics Research Group, Department of Immunology, Microbiology and Parasitology, Faculty of Science and Technology, University of the Basque Country (UPV/EHU), Leioa, Spain; Leibniz-Institut fur Naturstoff-Forschung und Infektionsbiologie eV Hans-Knoll-Institut, GERMANY

## Abstract

The filamentous fungus *Lomentospora (Scedosporium) prolificans* is an emerging opportunistic pathogen associated with fatal infections in patients with disturbed immune function. Unfortunately, conventional therapies are hardly of any use against this fungus due to its intrinsic resistance. Therefore, we performed an integrated study of the *L*. *prolificans* responses to the first option to treat these mycoses, namely voriconazole, with the aim of unveiling mechanisms involved in the resistance to this compound. To do that, we used a wide range of techniques, including fluorescence and electron microscopy to study morphological alterations, ion chromatography to measure changes in cell-wall carbohydrate composition, and proteomics-based techniques to identify the proteins differentially expressed under the presence of the drug. Significantly, we showed drastic changes occurring in cell shape after voriconazole exposure, *L*. *prolificans* hyphae being shorter and wider than under control conditions. Interestingly, we proved that the architecture and carbohydrate composition of the cell wall had been modified in the presence of the drug. Specifically, *L*. *prolificans* constructed a more complex organelle with a higher presence of glucans and mannans. In addition to this, we identified several differentially expressed proteins, including Srp1 and heat shock protein 70 (Hsp70), as the most overexpressed under voriconazole-induced stress conditions. The mechanisms described in this study, which may be directly related to *L*. *prolificans* antifungal resistance or tolerance, could be used as targets to improve existing therapies or to develop new ones in order to successfully eliminate these mycoses.

## Introduction

The filamentous fungus *Lomentospora prolificans*, formerly known as *Scedosporium prolificans* [1], is an opportunistic pathogen producing mycoses with a wide range of clinical manifestations, from superficial to deep infections. Importantly, when patients suffer from deep immunosuppression, *L*. *prolificans* tends to disseminate through the bloodstream due to its capacity to give rise to conidia inside body fluids and tissues, this infection pattern being the most dangerous and fatal [2].

As a consequence of the increase in the number of immunocompromised population over the last few decades, the prevalence of this species, along with the genus *Scedosporium*, is rising in several countries [3,4]. Thus, these fungi are becoming relevant emerging pathogens. In addition, these species are typically recovered from the respiratory tracts of cystic fibrosis patients, *L*. *prolificans* being among the most prevalent species in Australian [5,6] and Spanish [7] clinical settings. However, the most important feature of *L*. *prolificans* is its inherent resistance to virtually all currently available antifungal compounds, showing very low susceptibility patterns [8]. In consequence, infections caused by this species are associated with high morbidity and mortality rates [9].

Among the main classes of systemic antifungal drugs, azoles have been shown to be the most effective against *L*. *prolificans*, especially voriconazole (VRC), either alone or in combination with other compounds, such as terbinafine [10]. In fact, this triazole is the first-line treatment, as well as surgical debridement of the infected tissue when possible [11]. However, MICs observed in *in vitro* studies are unacceptably high (4->16 μg/ml) [8], with these concentrations in patients being difficult to achieve [9]. Moreover, although clinical response to treatment with VRC is better in comparison to other antifungal drugs, such as polyenes or echinocandins, disease outcomes are still poor [12,2]. Therefore, there is an increasing need for the development of new methods to manage *L*. *prolificans* infections.

In this sense, the cell wall represents one of the most interesting target organelles in fungal cells to help extend the range of therapeutic strategies. On the one hand, its specific composition and structure, completely absent in mammalian cells, reduces drug-associated toxicity and side-effects on host cells. On the other hand, molecular targets that make up this structure are highly available for antifungal compounds. Consequently, the identification of cell wall structures or molecules involved in antifungal resistance or pathobiology can be proposed as novel targets for developing truly effective therapies against these mycoses.

Consequently, in this work, we performed an integrated study of the changes occurring in *L*. *prolificans* cells when exposed to VRC. More specifically, changes in the cell wall architecture and composition were analysed, with special attention paid to the cell surface carbohydrates and subproteome. We were therefore able to identify several mechanisms and molecules that may be related to VRC resistance in this pathogenic fungus, which can be tested as new targets in the treatment of *L*. *prolificans* infections.

## Materials and methods

### Fungal strain, culture conditions, and germination assays

The *L*. *prolificans* strain CECT 20842, which showed MIC > 16 μg/ml for VRC in a previous analysis in our laboratory, was the subject of this research. In order to determine *L*. *prolificans* germination rates, conidia from 7-day old cultured potato dextrose agar (PDA) (Pronadisa, Madrid, Spain) were obtained, these were filtered through gauze, and the cell density determined using a counting chamber. Then, 5×10^5^ spores/ml were inoculated in potato dextrose broth (PDB) (Pronadisa) in the presence of 2 or 4 μg/ml of VRC (Sigma-Aldrich, St Louis, MO, USA), and incubated for 9 h at 37°C, 120 rpm, adding the same volume of DMSO in control flasks. At hourly intervals, 200 μl samples were obtained and fixed with 20 μl of 10% formaldehyde. At least one hundred cells per experimental time and condition were counted, and their germination rates determined. Three assays with technical triplicates were performed to ensure statistical significance.

To measure the rest of the parameters considered in this study, *L*. *prolificans* cells were harvested by centrifugation (5 min, at 4,500 g) after 9 h of culture alone or in the presence of 2 μg/ml of VRC, except when noted, and washed twice with PBS (50 mM, pH 7.5).

### Cell morphology analysis and indirect determination of chitin content by calcofluor white staining

Fungal cells exposed for 9 h to 0, 2, or 4 μg/ml of VRC were air-dried on slides overnight at 37°C before fixation with acetone for 20 min. Next, calcofluor white (40 ng/ml) was added for 20 min, dye excess being washed away with three applications of PBS. Finally, cells were visualised by a Zeiss Axioskop fluorescence microscope (Oberkochen, Germany), and micrographs analysed by ImageJ software (http://imagej.nih.gov/ij/) to quantify the emitted fluorescence, and cell morphological parameters. Three biological replicates were performed, at least one hundred cells being examined under the microscope each time.

### Transmission electron microscopy

To visualize fungal tissue under the electron microscope, cells were fixed with 2% glutaraldehyde in Sörenson buffer (SB) for 4h, washed with 6% sucrose in SB and postfixed in 1% OsO_4_ for 1 h at 4°C. Cells were washed with SB and dehydrated through an acetone series. Next, samples were embedded using the Epoxy Embedding Medium kit (Sigma-Aldrich). Semithin sections were obtained using a Leica UCT Ultramicrotome (Leica, Wetzlar, Germany), and then stained with toluidine blue. Ultrathin sections were contrasted with uranyl acetate and lead citrate. Electron micrographs were obtained using a transmission electron microscope Philips CM120 at 100 kV (Koninklijke Philips Electronics, Amsterdam, The Netherlands). All incubations were performed at room temperature, except when noted. After image acquisition, measurements of the thickness of the cell wall layers were performed using the ImageJ software in at least 20 cells per condition, quantifying 10 areas of each cell [13].

### Compositional analysis of whole cell wall and cell surface carbohydrates

To determine VRC-induced variations in the composition of the cell wall, we analysed both the carbohydrate composition of the whole organelle and those linked to cell surface-associated proteins (CSP). To measure the former, cells were washed three times with cold distilled H_2_O, resuspended in 10 mM Tris-HCl (pH 8), and disrupted by using glass beads and a MillMix 20 bead beater (Tehtnica, Slovenia) for 20 min at 30 Hz. Supernatants were recovered, and centrifuged 5 min at 3,800 g, then pellet washed five times with cold distilled H_2_O. Next, acid-based degradation of polysaccharides was carried out, as previously reported [14]. Briefly, 75 μl of 72% (v/v) H_2_SO_4_ per mg of cell walls were added, samples being left at room temperature for 3 h. Then cold distilled H_2_O was added little by little to achieve 1 M H_2_SO_4_, with the samples transferred to sealed glass tubes later, and incubated at 100°C for 3 h. Afterwards, samples were cooled, neutralised using saturated Ba(OH)_2_, and centrifuged 5 min at 3,800 g.

To obtain CSP, intact fungal cells were boiled in extraction buffer (50 mM Tris-HCl pH 8.0, 0.1 M EDTA, 2% [w/v] SDS, 10 mM DTT) for 10 min, as previously reported by Pitarch and co-workers [15]. Then, H_2_SO_4_ was added to the samples to achieve an acid concentration of 1 M, followed by the protocol mentioned above, for carbohydrate chemical lysis from this step.

After carbohydrate extraction, a semi-quantitative determination of the content of the cell wall in rhamnose, arabinose, glucosamine, galactose, glucose, and mannose was carried out by high-performance anion-exchange chromatography with pulsed amperometric detection (HPAEC-PAD) using CarboPac PA-10 columns and the chromatography system Dionex-600 (Thermo Scientific, Rockford, IL, USA). Peak area values for each monosaccharide were normalized using cell wall dry weights and compared between VRC-treated and control cells.

### Analysis of the effect of cell membrane- and wall-disturbing agents

*L*. *prolificans* conidia were obtained as previously mentioned and fungal cell suspensions adjusted to 10^7^ conidia/ml. Two μl of 1/10 serial dilutions were spotted onto PDA agar plates containing different concentrations of either VRC (0, 2 or 4 μg/ml), SDS, calcofluor white and congo red (0, 100, 250, 500, 750 or 1000 μg/ml), or a combination of VRC with each stress-inducing agent, as described elsewhere [16,17]. After two days at 37°C the plates were examined and photographed so that any fungal growth could be analyzed.

### Protein extraction and proteomic profiles of whole cell and cell surface-associated proteins

In order to identify proteins related to antifungal drug resistance, proteomes and surfaceomes of *L*. *prolificans*, exposed or not to VRC, were analysed as previously reported by our research group [18]. Briefly, to obtain whole cell or CSP protein extracts, fungal cells were, as mentioned above, disrupted or boiled in extraction buffer, respectively, with the proteins being precipitated by the TCA/Acetone method, and resuspended in rehydration buffer. Protein concentration was determined using the Pierce 660 nm Protein Assay Reagent (Thermo Scientific).

Proteomic profiles of both entire cells and CSP were resolved by two-dimensional electrophoresis (2-DE). First, 400 μg of fungal proteins dissolved in rehydration buffer were loaded onto 18-cm-long Immobiline DryStrip pH 3–10 (GE Healthcare) with 1% (v/v) 2-mercaptoethanol, 1% (v/v) ampholytes, and 0.002% (w/v) bromophenol blue for isoelectric focusing (IEF). After an overnight rehydration step, IEF was performed as follows: 500 V for 2,000 Vhr, 1,000 V for 9,000 Vhr, 8,000 V for 20,000 Vhr, and 8,000 V for 100,000 Vhr; 50 μA per strip. Then, two 15 min-long incubation steps in equilibration buffer (6 M urea, 75 mM Tris-HCl pH 6.8, 25.5% [v/v] glycerol, 2% [w/v] SDS, 0.002% [w/v] bromophenol blue) were performed, one with 1% (w/v) DTT and the other with 2.5% (w/v) iodoacetamide. Next, samples were separated by SDS-PAGE in a PROTEAN II xl Cell (Bio-Rad, Hercules, CA, USA) at 20 mA per gel, under refrigerated conditions. After that, fungal proteins were stained with Coomassie Brilliant Blue (CBB) and then gel images were acquired and analysed by using the ImageScanner III (GE Healthcare) and ImageMaster 2D Platinum Software (GE Healthcare), respectively.

### Identification of differentially expressed proteins by mass spectrometry

The protein spots most differentially expressed were manually excised and submitted for protein identification by LC-MS/MS, performed exactly as described elsewhere [18]. Briefly, selected SDS-PAGE spots were in-gel digested using trypsin (Roche, Basel, Switzerland) and the peptide mixture was analysed using a SYNAPT HDMS mass spectrometer (Waters, Milford, MA) interfaced with a nanoAcquity UPLC System (Waters). Obtained spectra were processed using VEMS [19] and searched for against the NCBI non-redundant (nr) database restricted to Fungi (version 20150309) using the online MASCOT server (Matrix Science Ltd., London; http://www.matrixscience.com). Even though the *L*. *prolificans* genome has been released it is not yet available publicly [20], so protein identification was performed by comparison with orthologous proteins from other fungi whose genomes were already available in the NCBInr database.

### Statistical analyses

Statistical analysis was performed using the GraphPad Prism 5 software (GraphPad Software, CA, USA). Differences in the mean were analysed by using the Student’s paired or unpaired *t*-test.

## Results

### Voriconazole-induced modifications on *L*. *prolificans* growth and morphology

**Fig 1:**
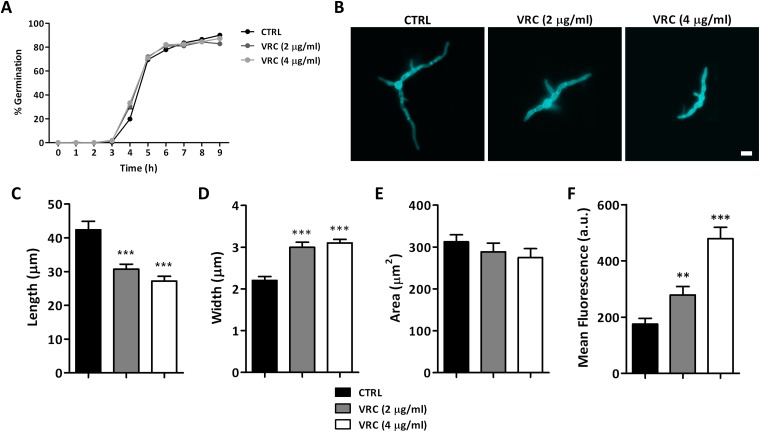
Morphological changes on *Lomentospora prolificans* cells caused by Voriconazole (VRC) exposure. Germination assays (A) were performed to analyze the effect of VRC on fungal cells. After 9 h of incubation cells were stained with calcofluor white and microscopically analysed (B) to determine their length (C), width (D), occupied area (E), and emitted fluorescence (F). Scale bar = 5 μm. Results are shown as mean ± SEM, n = 4. **p<0.01, ***p<0.0001 compared to non-treated cells. a.u., arbitrary units.

The exposure of *L*. *prolificans* to VRC did not affect fungal germination rates as hyphae started to grow 3 h after inoculation and reached approximately 80% of germinated cells in less than 8 h, in all the concentrations assessed (Fig 1A). In contrast, hyphal morphology was drastically modified, as visualised microscopically (Fig 1B), showing that exposure to the antifungal drug promoted a significant dose-dependent cell thickening and shortening (Fig 1C and 1D). Consequently, the total area occupied by fungal cells was not significantly modified (Fig 1E).

Moreover, calcofluor white allows the chitin content in fungal cells to be measured indirectly. So, the results show that VRC-treated cells emitted more fluorescence than the controls, this parameter being dependant on the VRC concentration; when the higher concentration was applied it rose to a level that was more than two times higher (Fig 1F).

### Ultrastructural and compositional changes on *L*. *prolificans* cell wall caused by voriconazole

**Fig 2:**
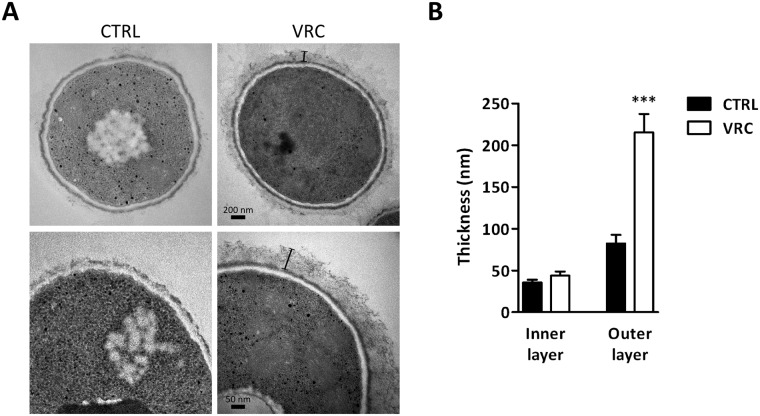
Ultrastructural analysis of voriconazole-induced changes on *Lomentospora prolificans* cells. Transmission electron microscopy images (A) and measurements of cell wall thickness (B) of non-treated and 2 μg/ml voriconazole-treated fungal cells. Black lines highlight the thickness of the outer fibrillar layer. Results are shown as mean ± SEM, n ≥ 20 cells. ***p<0.0001 compared to non-treated cells.

Transmission electron microscopy was used to determine the structural changes in the fungal cell wall under VRC-induced stress conditions. Thus, it was observed that cells treated with the antifungal drug increased their overall cell wall thickness (Fig 2A), this fact is, above all, due to a remarkable increase—almost three times—in the thickness of the fibrillar outer layer. However, no significant changes were observed in the low electro-dense inner layer between treated and untreated *L*. *prolificans* cells (Fig 2B).

**Fig 3:**
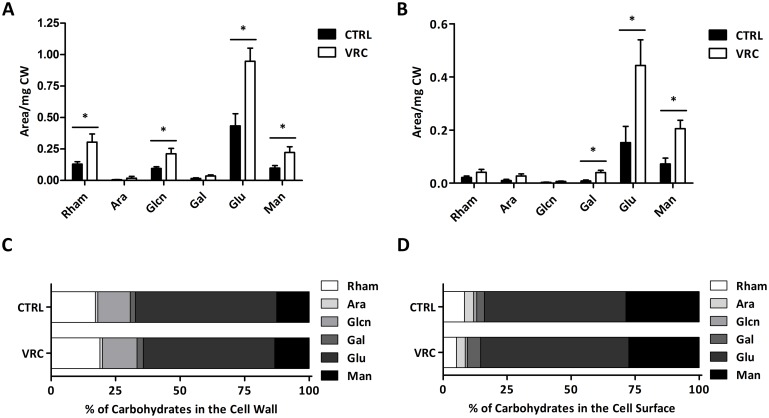
Biochemical characterization of the carbohydrate composition of *Lomentospora prolificans* cell wall. Carbohydrate compositional analysis of whole cell wall (A) and cell wall surface (B) upon exposure to 2 μg/ml voriconazole. Results are shown as mean ± SEM, n = 3. *p<0.05 compared to non-treated cells. Percentage of monosaccharide content in the whole cell wall (C) and surface (D).

On the other hand, the cell wall carbohydrate compositional analysis showed an overall increase in carbohydrate abundance in VRC-treated cells in comparison with controls. Precisely, exposure to VRC induced changes in several monosaccharides in cell wall backbone, with a significant increase in the rhamnose, glucosamine, glucose, and mannose content (Fig 3A). Moreover, when carbohydrate residues of CSP were scrutinised the rise was significant in the cases of galactose, glucose, and mannose residues (Fig 3B). Remarkably, in both extracts the most relevant changes occurred in the amounts of glucose and mannose. It is also worth highlighting the fact that, although there were significant differences in the relative abundance of the abovementioned specific monosaccharides, the increase in carbohydrate content was proportional between control and antifungal drug-treated cells (Fig 3C and 3D).

### Phenotypic analysis of the effect of voriconazole under cell membrane and wall stress conditions

**Fig 4:**
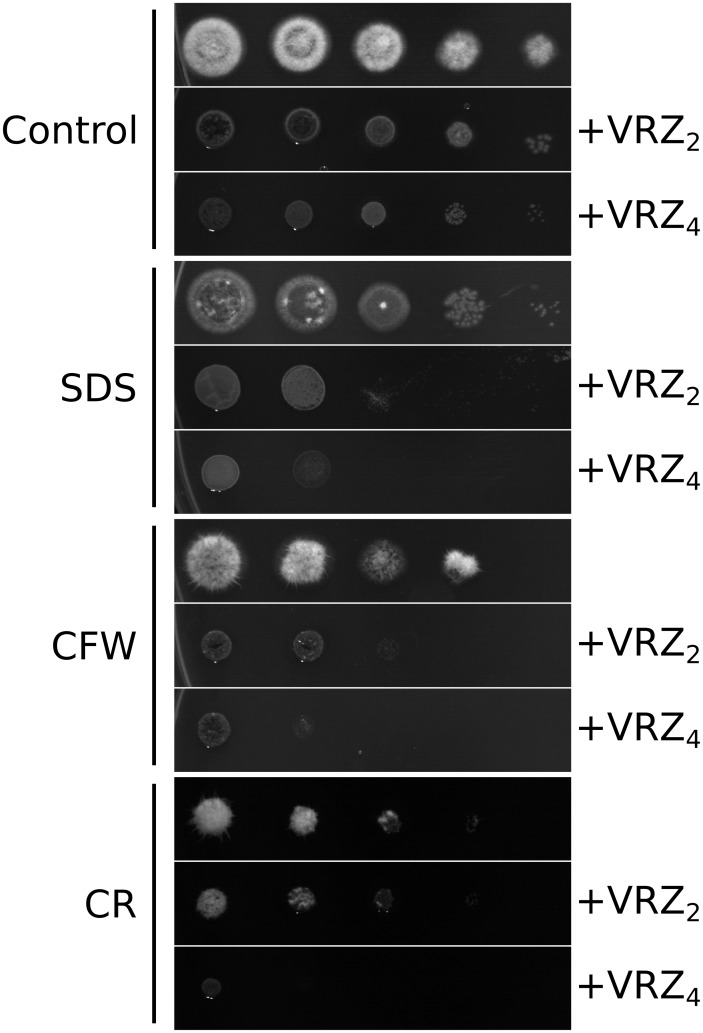
Effect of cell membrane- and wall-disturbing agents on *Lomentospora prolificans* in the presence of voriconazole. Decimal dilutions of conidial suspensions were spotted onto potato dextrose agar plates containing SDS (100 μg/ml), calcofluor white (500 μg/ml; CFW) or congo red (750 μg/ml; CR), and combined with 0, 2 or 4 μg/ml of voriconazole (VRC).

We performed *L*. *prolificans* spot assays in the presence of 100, 250, 500, 750 or 1000 μg/ml of the membrane-disturbing detergent SDS, and wall-stressing agents calcofluor white and congo red to analyze the susceptibility of the fungus to these compounds (S1 Fig). Then the effect of VRC during fungal cell membrane and wall stress conditions was evaluated by combining the antifungal drug with each of the agents mentioned above. Interestingly, it was observed that the presence of any of the three disturbing agents increased the effect of VRC on *L*. *prolificans* cells, its colonies showing lower levels of viability and changes in the morphology of the colonies when compared to the controls (Fig 4).

### Differential protein abundance in *L*. *prolificans* cell surface-associated proteins induced by voriconazole

**Fig 5:**
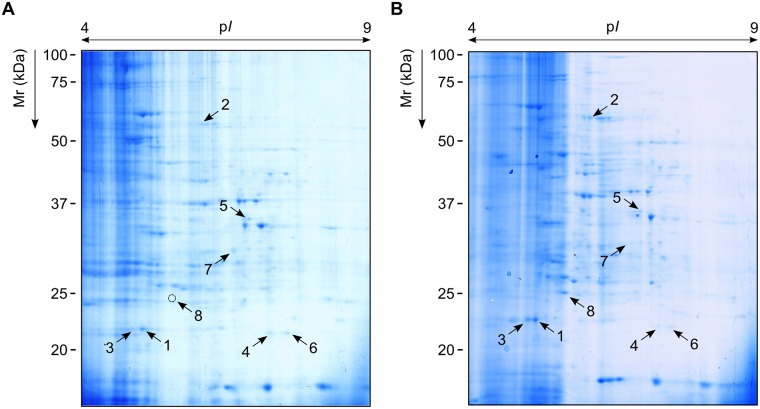
Effect of voriconazole on proteomic profiles of the *Lomentospora prolificans* cell surface subproteome. Fungal cells were grown in absence (A) or presence (B) of 2 μg/ml voriconazole, and their surfaceomes resolved by two-dimensional electrophoresis. Arrows point to the most differentially expressed protein spots that were identified by LC-MS/MS (See Table 1).

Finally, we analysed the quantitative and qualitative changes occurring in fungal proteomes and surfaceomes in response to VRC. Regarding the entire cell proteome, no significant changes, either quantitative or qualitative, were observed when control and VRC-exposed cells were compared (data not shown). Conversely, when surfaceomes were analysed up to seventy-two protein spots were found to be differentially expressed between treated and non-treated cell surfaceomes. Of these, forty protein spots were underexpressed and thirty-two overexpressed in VRC-treated cells, one of these only present after VRC exposure (Fig 5).

In order to focus on the most relevant proteins, eight protein spots were then identified: the four most overexpressed (including the one only present in VRC-treated surfaceomes), and the four most underexpressed in cells exposed to VRC, that is in comparison to control cells (Table 1). From the first group, two protein spots were identified as heat shock protein (Hsp) 70, one as a glutamate dehydrogenase-like protein, and another one as a Srp1 family-like protein (Serine-rich RNA polymerase I suppressor protein), the latter being the protein spot that only appeared when cells were exposed to VRC. Regarding the second group of protein spots, two of them correspond to 60S ribosomal protein-like protein, and the other two were identified as an inconclusive mixture of several proteins, including glyceraldehyde-3-phosphate dehydrogenase or RNA recognition domain-containing protein.

**Table 1:** Identification of the most differentially expressed proteins on *Lomentospora prolificans* cells exposed to voriconazole.

Spot No.	Ratio	NCBI No.	Protein name	Orthologous to	Matching peptides	Sequence coverage (%)	MASCOT score
**1**	3.365 (↑VRC)	gi|666870829	Heat shock 70 kDa protein	*Scedosporium apiospermum*	24	18	941
**2**	2.504 (↑VRC)	gi|666862145	Glutamate dehydrogenase-like protein (hypothetical protein SAPIO_CDS10451)	*Scedosporium apiospermum*	23	39	1073
**3**	2.312 (↑VRC)	gi|666870829	Heat shock 70 kDa protein	*Scedosporium apiospermum*	16	19	836
**4**	0.350 (↓VRC)	gi|666862378	60S ribosomal protein-like protein (hypothetical protein SAPIO_CDS9923)	*Scedosporium apiospermum*	13	10	286
**5**	0.322 (↓VRC)	gi|310798588	RNA recognition domain-containing protein	*Colletotrichum graminicola M1*.*001*	12	22	358
		gi|576039574	Malate dehydrogenase-like protein	*Chaetomium thermophilum var*. *thermophilum DSM 1495*	10	30	302
		gi|88766385	G-protein β subunit	*Metarhizium anisopliae*	11	19	300
**6**	0.285 (↓VRC)	gi|666862378	60S ribosomal protein-like protein (hypothetical protein SAPIO_CDS9923)	*Scedosporium apiospermum*	23	13	292
**7**	0.231 (↓VRC)	gi|380482569	Glyceraldehyde-3-phosphate dehydrogenase	*Pestalotiopsis fici* W106-1	12	21	411
		gi|666862386	D-arabinitol dehydrogenase-like protein (hypothetical protein SAPIO_CDS9932)	*Scedosporium apiospermum*	24	28	408
**8**	Unique in VRC	gi|302912421	Srp1 family-like protein (hypothetical protein NECHADRAFT_102628)	*Nectria haematococca* mpVI 77-13-4	47	42	572

## Discussion

The most challenging characteristic presented by *L*. *prolificans* is its inherent resistance to all currently available systemically active antifungal drugs [8], infections caused by this fungus being very difficult to manage and to eliminate. Of all the available antifungal drugs, the most effective agent against *L*. *prolificans* cells is VRC which, in combination with terbinafine, brings better results [10], suggesting that disturbing fungal cells in several ways may enhance antifungal drug activities [21]. However, up to now clinical results are not satisfactory and consequently there is an urgent need for novel therapeutic approaches that help to eliminate the fungus. Therefore, since the molecular basis of this species multidrug resistance is completely unknown, the aim of this work was to analyze the changes on *L*. *prolificans* cells when exposed to the antifungal drug VRC.

First, we observed by microscopy that while fungal germination rate did not alter after VRC exposure at any concentration assessed, hyphal morphology was dramatically modified with cells being significantly shorter and thicker when exposed to the antifungal drug. Moreover, calcofluor white staining showed that VRC-treated cells emitted a more intense fluorescence, this has been linked previously to an increase in chitin content [22]. These findings indicate that VRC could bring about changes in the fungal cell wall that may be associated with *L*. *prolificans* resistance.

Therefore, we looked deeper into the alterations in the structure and composition of the cell wall after exposure to VRC. Interestingly, using electron microscopy we determined that in *L*. *prolificans*, while the inner layer of this organelle did not significantly change, the thickness of the outer fibrillar layer after VRC exposure increased almost three times. In this respect, similar observations have been reported in *A*. *fumigatus* in response to nikkomycin Z or micafungin, either alone or in combination [23]. In addition, compositional analysis by ion chromatography revealed that VRC-treated cells presented an overall increase in the carbohydrate content in the cell wall, showing significantly higher levels of rhamnose, glucosamine, glucose, and mannose in the cell wall backbone, while in the cell surface those that increased significantly were galactose, glucose, and mannose. Remarkably, these alterations may be related to an increased presence of carbohydrate polymers, such as chitin, α- or β-glucans, and (rhamno)-mannans, which are the main components of the cell wall structure of many fungi, including this species [24]. As discussed previously, the increase in chitin abundance in VRC-treated cells was also proved by calcofluor white staining. Interestingly, stimulation of chitin synthesis in *C*. *albicans* increases echinocandin tolerance/resistance [25], and strains with intrinsic higher abundance in this polysaccharide have been related to lower susceptibility to these antifungal drugs [26]. However, to our knowledge, the other compositional changes observed in this study have not so far been reported for any antifungal drug-induced effect. Taking all these data together, it can be concluded that VRC causes dramatic changes in both the cell wall structure and its composition, which might be due to cell membrane or wall instability promoted by the antifungal drug, as previously proposed for the effect of fluconazole on *C*. *albicans* [17]. Membrane instability after VRC treatment was proved when *L*. *prolificans* was grown in the presence of SDS combined with the antifungal drug. The addition of non-lethal concentrations of the detergent to the culture medium increased the sensitivity of the fungus to VRC, causing a loss of viability and important morphological changes in growing colonies. In addition, the cell wall-perturbing agents calcofluor white and congo red also brought about noteworthy changes in the colonies and viability of *L*. *prolificans* when combined with the triazole. These results highlight the relevance of the fungal cell wall modifications in VRC resistance and, a still unproven relation to a protective mechanism developed by the fungus to cope with VRC, e.g. by reducing osmotic stress, or impeding and decreasing the entry of the antifungal drug in *L*. *prolificans* cells.

Finally, to determine whether VRC-induced changes were occurring in whole proteomes or cell surface-associated proteins, a proteomics-based study of *L*. *prolificans* was performed. Over the last few years, these approaches have been helpful in identifying both novel and specific targets, and to describe the molecular interactions between drugs and fungi, as previously reported for *C*. *albicans* [17,27], *Cryptococcus gattii* [28], or *Aspergillus fumigatus* [29,30]. Surprisingly, our results showed no significant differential protein abundance between VRC-treated and non-treated cell whole proteomes. This unexpected finding may be due to several factors, including some related to the experimental paradigm used in this study or to limitations in the proteomic approach employed, which might hamper the detection of low-abundant proteins. On the contrary, a total of seventy two protein spots were found, either up- or down-regulated, when only cell surface subproteomes were scrutinised. Among these, Hsp70 (in two protein spots), glutamate dehydrogenase-like protein, and a Srp1 family-like protein (unique in VRC-induced subproteome), were identified as up-regulated; and 60S ribosomal protein-like protein (in two protein spots), and two protein spots which could only be identified as a mixture of proteins, as down-regulated. The fact that protein identification had to be carried out by searching against orthologous sequences available in the databases may have hampered a more accurate identification of these proteins.

The interest in heat shock proteins (HSP), especially Hsp90, as antifungal targets has risen in the last years [31]. Beyond their role as chaperones to stabilize other protein structures in thermal shock conditions, HSPs have been related to the orchestration of antifungal drug resistance, or tolerance, under the regulation of calcineurin, which has many roles in fungal cell growth, virulence, and stress resistance [32]. In fact, Hsp70, which is also involved in the abovementioned Hsp90-calcineurin pathway, has recently been associated with amphotericin B and caspofungin resistance in *A*. *terreus* and *A*. *fumigatus*, respectively [33,34]. Therefore, our results are in accordance with these recent reports, suggesting that this protein could be significant in *L*. *prolificans* VRC resistance. Furthermore, this protein has been identified previously as an important *L*. *prolificans* antigen that is recognised by immunocompetent individuals, by both immunoglobulin (Ig) A and G, and is one of the most abundant and seroprevalent antigens [18,35,36].

On the other hand, a putative Srp1 protein was identified, this protein spot being only detected in VRC-treated cells. Although little is known about these proteins, they are putative suppressors of RNA polymerases [37], which are normally located in the nuclear membrane. However, they have been also detected in the fungal cell wall [38] and linked to cell thermal stress [39]. Regarding the 60S ribosomal proteins that were found down-regulated upon VRC exposure, these are involved in protein translation, as structural parts forming ribosomes. Interestingly, they have been associated with antifungal stress by either being down- [40] or over-expressed [28], which may be due to differences in the mechanisms pathways of action used by the drugs. It is worth highlighting the fact that both Srp1 and 60S ribosomal proteins are related to protein synthesis, and may be related to the morphological changes occurring in *L*. *prolificans* cells in response to VRC. Finally, proteins related to amino acids such as glutamate dehydrogenase, and the carbohydrate metabolism (such as malate dehydrogenase, in the protein spots with a mixture of proteins) have been found to be differentially down-regulated, showing that the fungal cell is regulating its own metabolism.

In summary, the present work has extensively analysed the biological responses of *L*. *prolificans* in the presence of VRC, above all focusing on the molecular changes occurring in the fungal cell wall. Precisely, changes in cell shape after exposure to VRC have been described, *L*. *prolificans* forming shorter and wider cells compared to untreated ones. Moreover, crucial modifications in the cell wall structure and carbohydrate composition have also been noted, the fungus building a thicker cell wall with a higher level of glucans and mannans, among other carbohydrates. In addition, several CSP were also found to be altered, showing the relevance of Hsp70 and Srp1 as they were identified as the most overexpressed proteins after VRC exposure. Targeting these processes or specific proteins may be helpful for the development of novel and effective therapies against mycoses caused by *L*. *prolificans*, improving the health of the patients suffering from them.

## Supporting information

S1 Fig Susceptibility of *Lomentospora prolificans* to cell membrane- and wall-disturbing agents. Decimal dilutions of conidial suspensions were spotted onto potato dextrose agar plates containing 0, 100, 250, 500, 750 or 1000 μg/ml of SDS, calcofluor white (CFW) or congo red (CR).
(TIF)
