# Supplementary figures and images for: Molecular and cellular responses of the pathogenic fungus Lomentospora prolificans to the antifungal drug voriconazole
Source: PLoS One. 2017 Mar 31;12(3):e0174885. doi: 10.1371/journal.pone.0174885 (PMC5376303; doi:10.1371/journal.pone.0174885)

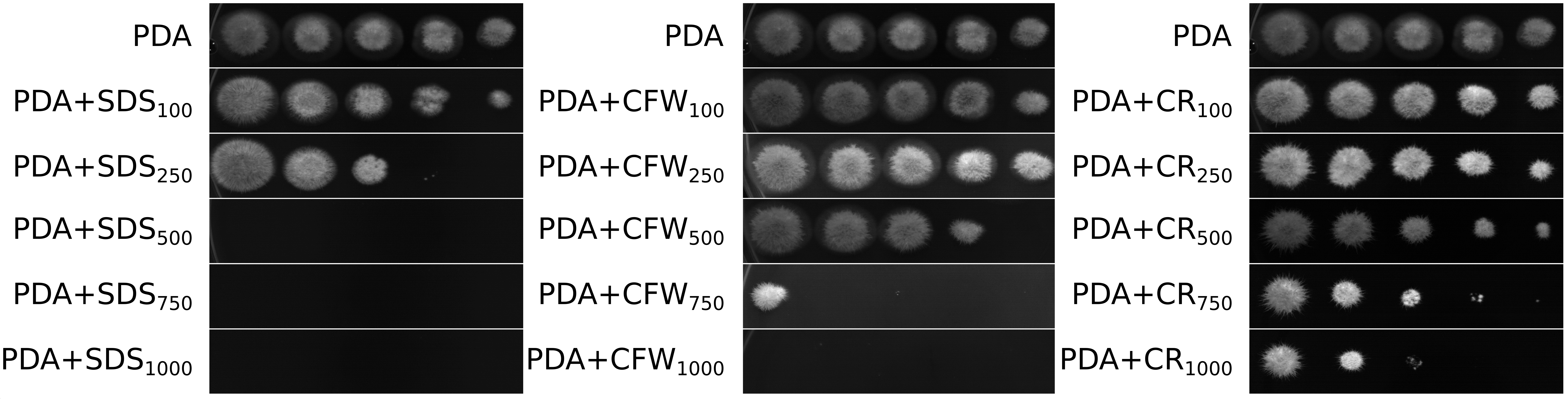

Supplement: S1 Fig — Decimal dilutions of conidial suspensions were spotted onto potato dextrose agar plates containing 0, 100, 250, 500, 750 or 1000 μg/ml of SDS, calcofluor white (CFW) or congo red (CR). (TIF) [file pone.0174885.s001.tif]
